# Germline Genetic Association between Stromal Interaction Molecule 1 (STIM1) and Clinical Outcomes in Breast Cancer Patients

**DOI:** 10.3390/jpm10040287

**Published:** 2020-12-17

**Authors:** Chi-Cheng Huang, Min-Rou Lin, Yu-Chen Yang, Yu-Wen Hsu, Henry Sung-Ching Wong, Wei-Chiao Chang

**Affiliations:** 1 Comprehensive Breast Health Center, Taipei Veterans General Hospital, Taipei 11217, Taiwan; chishenh74@gmail.com; 2 College of Medicine, Taipei Medical University, Taipei 11031, Taiwan; cheney_yaung@hotmail.com; 3 School of Public Health, College of Public Health, National Taiwan University, Taipei 10617, Taiwan; 4 Department of Surgery, Cathay General Hospital, Taipei 106, Taiwan; 5 Department of Clinical Pharmacy, School of Pharmacy, Taipei Medical University, Taipei 11031, Taiwan; jennielmr@gmail.com (M.-R.L.); miningyue@gmail.com (H.S.-C.W.); 6 Ph.D. Program for Translational Medicine, College of Medical Science and Technology, Academia Sinica, Taipei Medical University, Taipei 11031, Taiwan; fish770426@hotmail.com; 7 Department of Medicine, The University of Chicago, Chicago, IL 60611, USA; 8 Master Program for Clinical Pharmacogenomics and Pharmacoproteomics, School of Pharmacy, Taipei Medical University, Taipei 11031, Taiwan; 9 Integrative Research Center for Critical Care, Wan Fang Hospital, Taipei Medical University, Taipei 11031, Taiwan; 10 Department of Medical Research, Shuang Ho Hospital, Taipei Medical University, New Taipei 23561, Taiwan

**Keywords:** breast cancer, *STIM1*, genetic variants

## Abstract

Among all cancers in women, breast cancer has the highest incidence. The mortality of breast cancer is highly associated with metastasis. Migration and malignant transformation of cancer cells have been reported to be modulated by store-operated calcium (SOC) channels, which control calcium signaling and cell proliferation pathways. Stromal interaction molecule 1 (*STIM1)* is a calcium sensor in the endoplasmic reticulum, triggering the activation of store-operated calcium signaling. However, the clinical relevance of *STIM1* in breast cancer is still unclear. Here, we recruited 348 breast cancer patients and conducted a genetic association study to address this question. Four tagging germline single nucleotide variants (SNVs) in *STIM1* were selected and RNA sequencing data of 525 breast cancer samples from The Cancer Genome Atlas (TCGA) database were evaluated. The results show that rs2304891 and rs3750996 were correlated with clinical stage of breast cancer. Expression quantitative trait loci (eQTL) analysis indicated that risk G allele of *STIM1* contributed to the higher expression of *STIM1*. In addition, we found an increased risk of rs2304891 G allele and rs3750996 A allele in estrogen receptor (ER) positive and progesterone receptor (PR) positive patients. In conclusion, our results suggest that germline SNV, rs2304891 and rs3750996 as well as *STIM1* expression are important biomarkers for the prediction of clinical outcomes in breast cancer patients.

## 1. Introduction

Breast cancer has the highest incidence in females around the world [1], and it is the fourth leading cause of cancer deaths in Taiwan [2]. Currently, molecular histology is applied to characterize breast cancer subtypes according to the presence of estrogen receptor (ER), progesterone receptor (PR) and human epidermal growth factor II (HER-2). Following radiotherapy and surgery, targeted therapies including anti-hormone and anti-HER-2 antibodies can be administered to patients based on specified cancer subtypes [3,4,5]. Primary breast tumor treatments can effectively delay disease progression and prolong overall survival. However, the occurrence of tumor metastasis in distant organs compromises treatment efficacy and constitutes the major cause of death among breast cancer patients [6].

Breast cancer is heterogeneous in terms of molecular aberrations. The oncogenesis originates from single nucleotide variants (SNVs), chromosomal DNA copy number variations and manifests through transcription and translation as gene/protein expression alternations [7]. Genetic variations not only provide a plethora of sources of prognostic biomarkers but pave a way to identify potential therapeutics in terms of precision medicine. For instance, poly ADP ribose polymerase (PARP) inhibitors are one of the reasonable choices for breast cancer with deleterious germline *BRCA1/2* mutations through synthetic lethargy [8]. Additionally, previous studies have widely reported that calcium signaling is critical in tumor metastasis [9]. As a secondary messenger, calcium modulates a wide variety of intracellular responses including enzymatic binding and protein–protein interaction [10], vesicle transport [11], immune response to pathogen [12], cell motility, and tumor metastasis [13]. Thus, the changes of calcium concentration by calcium pumps and channels in different cellular compartments are essential in a precise, time-dependent manner. Among all calcium channels on cell membranes, the store-operated calcium (SOC) channel efficiently refills and maintains intracellular calcium concentrations in the endoplasmic reticulum (ER) after calcium release into the cytosol. Stromal interaction molecule 1 (*STIM1*) of the ER was identified as an calcium sensor that regulates the activation of the SOC channel. When the intracellular calcium concentration is low [14,15,16], *STIM1* activates the SOC channel [17,18], which in turn, triggers calcium influx [19]. SOC channel-mediated calcium signaling is essential for tumor cell migration and invasion especially in breast cancer [13], melanoma [20], glioma [21], and colorectal cancer (CRC) [22]. In addition, inhibition of the SOC channel was found to reduce cell proliferation [23], cancer cell migration [24,25], and inflammatory reactions [26,27].

*STIM1* overexpression was found to increase cyclooxygenase-2 (COX-2) gene expression and prostaglandin E2 (PGE2) production, thereby promoting tumor development and cell migration in CRC [22]. Although previous studies have revealed that *STIM1* gene expression is related to breast cancer progression [13,28], there is yet no evidence to indicate the role of *STIM1* genetic variants and cancer. Thus, the aim of the current study was to investigate the correlation between genetic variants of *STIM1* and clinical outcomes of breast cancer.

## 2. Materials and Methods

### 2.1. Study Cohorts

In the present study, we enrolled 348 breast cancer (BC) samples of Han Chinese (Taiwanese) ancestry. The cases were confirmed by pathology in Cathay General Hospital (CGH), Taipei, Taiwan. Malignant tissues and peripheral blood were obtained during surgery or biopsy. Immunohistochemistry (IHC) was used to characterize pathological features of the breast cancer tissues, including the estrogen receptor (ER), progesterone receptor (PR), and human epidermal growth factor receptor-2 (HER2) status. Clinical stage was categorized as tumor-node-metastasis (TNM, classification of malignant tumors) based on the American Joint Committee on Cancer 7th edition [29]. The study has been approved by the institutional review board (IRB) of CGH, Taipei, Taiwan. All clinical specimens were obtained with the consent of patients.

As for public resources, RNA sequencing (RNA-seq) data of breast invasive carcinoma (BRCA) were downloaded from The Cancer Genome Atlas (TCGA) database [30] using the *TCGAbiolinks* package [31] in R. The data consisted of 1222 samples, of which 1102 were primary solid tumors, 113 were solid normal tissue, and 7 were metastatic samples. We defined a square symmetric matrix of Pearson correlation among all primary solid tumor samples, a correlation cutoff of 0.6 was set to identify possible outliers. To test the prognostic correlation of STIM1 expression, 525 tumor samples with available overall survival (OS) data were used to conduct survival analysis.

### 2.2. Selection of Germline Tagging Single Nucleotide Variants (SNVs)

The tagging SNVs of *STIM1* were selected with the criteria of: (1) Beijing Han Chinese (CHB) ancestry; (2) a linkage disequilibrium threshold (LD) of >0.8 (by *r*^2^) to the nearby SNVs; (3) a minor allele frequency (MAF) of ≥10% from HapMap project, and (4) located in exonic, 3′ untranslated region (3′UTR), or 5′ untranslated region (5′UTR) of *STIM1* gene. Four selected SNVs, including rs2304891 (exon 8), rs1561876 (3′UTR), rs3750994(3′UTR), and rs3750996 (3′UTR) were selected for genotyping. Genotype frequency of 4 SNVs was queried from the Taiwan Biobank (TWB; https://taiwanview.twbiobank.org.tw/index; accessed on 29 October 2020) and Ensembl genome database [32].

### 2.3. TaqMan Genotyping Assay

Genomic DNA was extracted from peripheral bloods with a Gentra Puregene^®^ kit (Qiagen, Hilden, Germany). After DNA isolation was completed, samples were analyzed for quality and quantity using a NanoDrop spectrophotometer, and all samples were normalized to a concentration of 10 ng/µL for genotyping analysis. Genotyping was carried out by a polymerase chain reaction (PCR) on an ABI 7500 instrument according to the manufacturer’s instructions (Applied Biosystems, Foster City, CA 94404 USA). TaqMan^®^ Genotyping Assays (Applied Biosystems, Foster City, CA 94404 USA) were applied in the PCR. The genotype of each patient was identified by StepOne software v2.3 (Applied Biosystems, Foster City, CA 94404 USA).

### 2.4. Statistical Analysis

R studio (with R version 4.0.2) was used for all analysis in this study. For the SNV analysis, association tests were conducted with *SNPassoc* package. The case-control sets and continuous data were analyzed for allele frequency differences for the four *STIM1* tagging SNVs using logistic regression and linear regression, respectively. For the regression model, patients’ age was included as covariate. A *p*-value of <0.05 was regarded as significant without multiple testing correction (MTC). Haplotype analysis in early- and late-stage Taiwanese BC patients was completed by the *haplo.stat* package, with an additive model. For the survival analysis, the Kaplan–Meier estimator (a non-parametric statistic for estimating survival function of TCGA BRCA patients) and log-rank test (as implemented in *survival* package) were used to depict the prognostic correlation of *STIM1* expression.

## 3. Results

### 3.1. Basal Characteristic of the Breast Cancer Patients

A total of 348 breast cancer cases were recruited in this study (Table 1). All patients were female with the mean age of 53.13 ± 11.17 years (range: 23–89 years). Among 295 BC patients with available ER status, 78.31% (*n* = 131) were ER positive (ER+). Moreover, 64.29% (out of 294 samples with available PR status) of BC patients were PR positive (PR+), and 66.55% (out of 293 samples with available HER2 status) of BC patients were HER2 positive (HER2+). Out of 283 BC samples with available clinical stage data, 48.76% (*n* = 138) were recruited at stage I, 34.28% (*n* = 97) at stage II, and 16.96% (*n* = 48) at stage III.

### 3.2. Association of STIM1 Genetic Variants with BC Disease Staging

**Figure 1:**
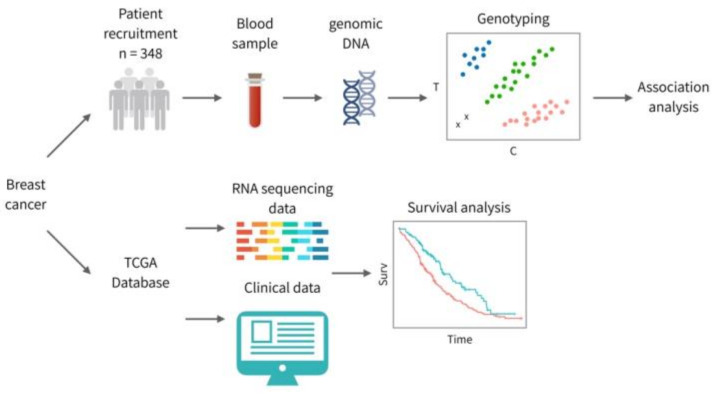
An overview of the study design; 348 BC patients were recruited in this study, and genomic DNA was extracted from peripheral blood samples. Four tagging single nucleotide variants (SNVs) were selected for genotyping and further examined for germline association of the stromal interaction molecule 1 (STIM1) gene. RNA-seq data and clinical data of BC were downloaded from The Cancer Genome Atlas (TCGA) database to conduct survival analysis.

**Figure 2:**
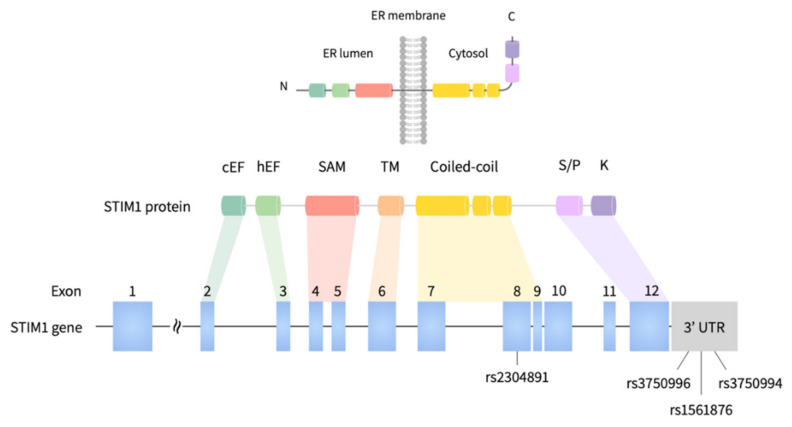
Graphical overview of the human *STIM1* gene and 4 tagging SNVs in this study.

The overall study workflow is shown in Figure 1. Results from patient recruitment and TCGA database will be analyzed. Four tagging SNVs (rs2304891, rs3750996, rs1561876 and rs3750994) were selected for genetic association study (Figure 2 and Table 2). The relationship between BC and *STIM1* polymorphisms was characterized. We classified TNM stage into early-stage (stage I and stage II) and late-stage (stage III). For rs2304891, carriers with the A/G (odds ratio = 2.49) as well as the G/G (odds ratio = 4.48) genotypes exhibited a higher risk in disease progression compared to patients with the A/A genotype (*p* = 5.17 × 10^−3^; genotypic model). Statistical significance can also be found in the dominant model (data not shown). Moreover, rs3750996 heterozygous A/G genotype was significantly correlated with a slower progression compared to the A/A genotype (*p* = 5.50 × 10^−4^, odds ratio = 0.35; genotypic model (Table 3). Importantly, statistical significance can still be found in the dominant and recessive model (data not shown)).

In addition, we found an increased risk of rs2304891 G allele (*p* = 9.18 × 10^−4^, odds ratio = 9.86; genotypic model) and rs3750996 A allele (*p* = 3.16 × 10^−3^, odds ratio = 0.28; genotypic model) in ER+ patients (Table 4). We also discovered a statistical significance of rs2304891 under the dominant model and rs3750996 under the dominant and recessive model (data not shown). In other words, ER+ patients who carrying a risk allele of rs2304891 or rs3750996 who happen to be ER+ are more likely to develop late-stage BC than others. Similar patterns can also be observed in PR+ patients (Supplementary Table S1). For the rs2304891 SNV, PR+ patients bearing the G allele had a significantly increased risk of disease progression compared to A allele carriers (odds ratio = 6.02 for A/G, and odds ratio = 10.51 for G/G; genotypic model). For the rs3750996 SNV, A is the risk allele that PR+ A/G carriers exhibited significantly lower risk than PR+ A/A carriers (odds ratio = 0.23 for A/G; genotypic model). Furthermore, the rs3750996 A/A genotype was also associated with an increased risk of disease progression in HER2+ BC patients (*p* = 0.0315, odds ratio = 0.38 for A/G; genotypic model; Supplementary Table S2).

### 3.3. Association of STIM1 Haplotypes with Disease Staging in BC Patient

**Figure 3:**
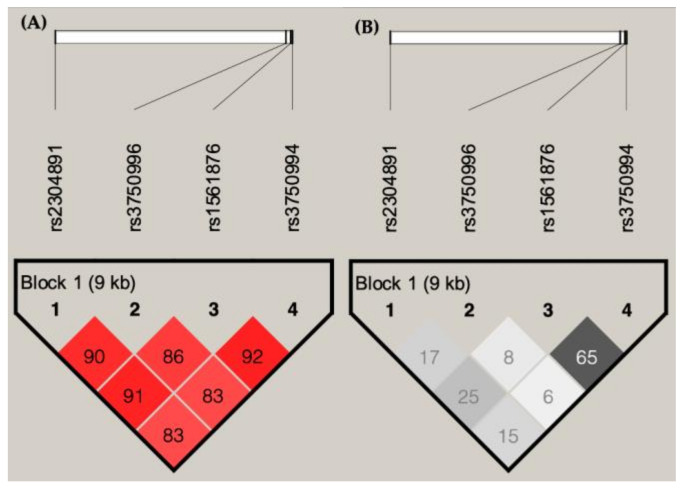
Linkage disequilibrium (LD) plots showing (**A**) D’ and (**B**) r^2^ of 4 SNVs of *STIM1* in breast cancer patients.

In order to have a comprehensive understanding of the most important haplotype of *STIM1*, an LD map was generated, using all four SNVs that are in high LD (D’ > 0.8; Figure 3). A haplotype analysis was then performed (Table 5). Patients with haplotypes of rs2304891/rs3750996/rs1561876/rs3750994 G/A/A/T have a higher risk of developing late-stage BC (*p* = 0.0028, odds ratio = 3.84).

### 3.4. Correlation between STIM1 Expression and the Survival of Breast Cancer Patients

**Figure 4:**
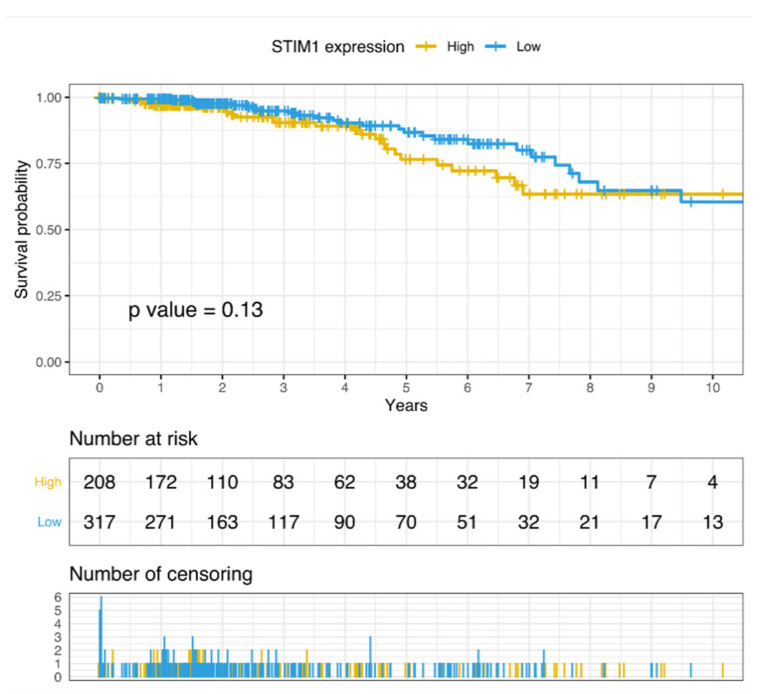
Survival analysis of high- and low-*STIM1* expression group of breast cancer patients in TCGA.

We further investigated whether *STIM1* gene expression was correlated with morbidity in BC patients. By leveraging TCGA RNA sequencing data of BC samples, *STIM1* expression was divided into the high expression group and low expression group by higher (*n* = 208) and lower (*n* = 317) quartile. According to the grouping, a Kaplan–Meier estimator was applied, and log-rank test was further conducted for survival analysis. Though no significant association was identified, a modest trend was observed that *STIM1* overexpression is associated with poorer survival (Figure 4).

### 3.5. eQTL Analysis of rs2304891 Confirmed the Correlations between Genotypes and Tissue-Specific Gene Expression Levels

To investigate the association between gene expression profiles and the genetic variants of *STIM1*, we queried the GTEx portal [33], which includes a variety of tissue expression quantitative trait loci (eQTLs). The results indicated that rs2304891 was significantly associated with the expression of the *STIM1* gene in the left heart ventricle and tibial nerve tissues (Supplementary Table S3), with a higher expression level in GG genotype compared to AA genotype (Supplementary Figure S1).

## 4. Discussion

In this study, we investigated the association between clinical relevance of breast cancer and genetic variants of *STIM1*. Results revealed that two variants (rs2304891 and rs3750996) are associated with the disease stage. We further identified the correlation between *STIM1* gene expression and clinical outcomes of breast cancer by using the TCGA database. A trend was observed between higher *STIM1* expression and poorer survival. Regarding the haplotype analysis, patients with the G/A/A/T (*p* = 0.0028, odds ratio: 3.84) genotypes tended to be in the later-stage cancers. Furthermore, eQTL analysis of rs2304891 confirmed that the risk GG genotypes was associated with the higher expression of *STIM1* although rs2304891 is a synonymous substitution. rs3750996 is located in the 3′UTR; however, no previous reports have indicated the effects of this SNV in *STIM1* gene expression. In miRBase, mutated rs3750996 was reported to change the binding affinity of microRNA223 (miR223) [34], but whether the microRNA223 influences *STIM1* gene expression still requires more investigations.

Several studies have reported the association between genetic polymorphisms in store-operated calcium signaling and human diseases [35,36,37,38]. For example, *STIM1* polymorphisms have been shown to associate with the inflammatory index (erythrocyte sedimentation rate and C-reactive protein) in ankylosing spondylitis patients. Recently, *STIM1* mutation was reported to influence T cell functions, which may increase the risk of hypertensive renal disease [39]. In addition to *STIM1*, genetic variants in another key component of store-operated calcium channel, *Orai1*, are critical for complex diseases, especially for inflammatory diseases. Based on the genetic association study, For example, *Orai1* polymorphisms have been shown to associate with the susceptibility for recurrence of calcium nephrolithiasis, Kawasaki disease and atopic dermatitis [36,37,40]. Here, our study revealed different findings. Genetic variants of *STIM1* associated with the development of breast cancer might be connected through the alteration of *STIM1* expression. Although a trend of correlation between *STIM1* expression and cancer survival was observed, the results did not reach statistical significance. *STIM*-mediated calcium signaling involves the immune regulation as well as cancer cell migration. Whether the roles of *STIM1* in the cancer microenvironment are tended to immune pathways or simply control cell cycle/cell growth is an important question to address in the future.

While our study illustrates the associations between *STIM1* genetic variants and clinical stage, we also found that patients with the risk alleles of rs2304891 or rs3750996 have a higher risk of late-stage breast cancer especially in ER+ or PR+ tumors. Since breast cancer is likely affected by hormones, cancer cells that express estrogen or progesterone receptors might increase the tumor growth. Tamoxifen is an FDA-approved selective ER modulator (SERM) that acts as an anti-estrogen in breast cells. Previous studies have shown that ER positive breast tumors generally have a better prognosis than ER negative tumors; however, patients actually display varying levels of responsiveness to therapy [41]. One explanation is inflammation [42]. Baumgarten et al. indicated that co-expression of estrogen and NF-kB-mediated inflammatory pathways can enhance chemoresistance [43]. The interaction between ER expression and NF-κB signaling could trigger breast cancer cells to an aggressive phenotype. In addition, store-operated calcium dependent regulation for NF-kB activation has been studied extensively. In cancer cells, the major calcium influx pathway is through store-operated calcium channels, which are composed by *STIM1* and *Orai1* [13,20,23]. Thus, the combination therapy against both NFkB-mediated inflammation and estrogen expression might be an effective therapy for ER positive patients. Additionally, markers of rs2304891 or rs3750996 would be very helpful to screen the higher risk ER+ breast cancer patients.

In conclusion, we found that rs3750996 and rs2304891 were associated with the progression of breast cancer. In addition, functional prediction by eQTL analysis further confirmed the effects of risk allele in gene expression. Indeed, overexpression of STIM1 has been shown to promote cancer progression [22]. Downregulation of STIM1 expression by miR-223 resulted in the inhibition of breast cancer invasion [44]. In light of the fact that *STIM1* has been reported to link to cellular migration of cancer cells, our results further indicate a clinical role of *STIM1* variants in the risk of breast cancer.

## Figures and Tables

**Table 1:** Basal characteristics of Taiwanese breast cancer (BC) patients.

Characteristic	Total No. = 348
Gender (female; no.)	348 (100)
Age (years old, mean ± sd (range))	53.13 ± 11.17 (23–89)
ER ^a^ (no.)	
Positive	231 (66.38)
Negative	64 (18.39)
n.a.	53 (15.23)
PR ^b^ (no.)	
Positive	189 (54.31)
Negative	105 (30.17)
n.a.	54 (15.52)
HER2 ^c^ (no.)	
Positive	195 (56.03)
Negative	98 (28.16)
n.a.	55 (15.80)
AJCC ^d^/TNM ^e^ stage (no.)	
1	138 (39.66)
2	97 (27.87)
3	48 (13.79)
n.a.	65 (18.68)

n.a., not available. ^a^ Estrogen receptor. ^b^ Progesterone receptor. ^c^ Human epidermal growth factor receptor 2. ^d^ American Joint Committee on Cancer. ^e^ Tumor-node-metastasis. There were 295, 294 and 293 patients with ER, PR and HER2 receptor information recorded, respectively.

**Table 2:** Genotype frequencies of STIM1 germline variants in different ethnic populations.

			Population Genotypic Frequency ^a^						
dbSNV ID	Genotype	Chr:Pos ^b^	AFR	AMR	EUR	SAS	EAS	TWB	Our Cohort
rs2304891	G/G	11:4103524	0.020	0.236	0.336	0.225	0.210	0.199	0.183
	A/G		0.156	0.496	0.463	0.503	0.464	0.481	0.477
	A/A		0.825	0.268	0.201	0.272	0.325	0.320	0.340
rs3750996	G/G	11:4113200	0	0	0	0.010	0.060	0.050	0.081
	A/G		0	0.003	0	0.147	0.315	0.339	0.325
	A/A		1	0.997	1	0.843	0.625	0.611	0.594
rs1561876	G/G	11:4113395	0.498	0.061	0.012	0.004	0.067	0.079	0.059
	A/G		0.415	0.326	0.209	0.182	0.331	0.381	0.422
	A/A		0.088	0.614	0.779	0.814	0.601	0.540	0.519
rs3750994	G/G	11:4113470	0	0.012	0.002	0	0.052	0.054	0.031
	T/G		0.015	0.205	0.056	0.055	0.282	0.323	0.350
	T/T		0.985	0.784	0.942	0.945	0.667	0.623	0.619

^a^ Data were collected from Taiwan Biobank (TWB) and Ensembl genome browser (release 94). TWB, Taiwan Biobank; AFR, Africa; AMR, America; EAS, East Asia; EUR, Europe; SAS, South Asia. ^b^ Based on genomic coordination hg37.

**Table 3:** Association analysis between germline genetic polymorphisms of *STIM1* and TNM stage in breast cancer patients.

dbSNV ID	Genotype	Late Stage ^a^ *N* (%)	Early Stage ^b^ *N* (%)	Genotype Model	
				OR (95% C.I.) ^c^	*p* Value
rs2304891	G/G	15 (31.9)	37 (16.4)	4.48 (1.73~11.62)	**5.17 × 10^−3^** *
	A/G	24 (51.1)	104 (46.2)	2.49 (1.06~5.86)	
	A/A	8 (17.0)	84 (37.3)	Reference	
rs3750996	G/G	0 (0)	19 (8.3)	0	**5.50 × 10^−4^** **
	A/G	8 (17.0)	75 (32.9)	0.35 (0.15~0.79)	
	A/A	39 (83.0)	134 (58.8)	Reference	
rs1561876	G/G	1 (2.3)	16 (7.3)	0.42 (0.05~3.40)	0.2631
	A/G	24 (54.5)	94 (42.9)	1.47 (0.76~2.87)	
	A/A	19 (43.2)	109 (49.8)	Reference	
rs3750994	G/G	2 (4.4)	10 (4.4)	1.24 (0.25~6.15)	0.9488
	T/G	17 (37.8)	81 (35.8)	1.08 (0.55~2.12)	
	T/T	26 (57.8)	135 (59.7)	Reference	

The *p* value was adjusted for age. * *p* value < 0.05 and ** *p* value < 0.005 are shown in bold. ^a^ Late-stage included stage III. ^b^ Early-stage included stages I and II. ^c^ Odds ratio (OR) and 95% confidence intervals (C.I.).

**Table 4:** Association analysis between germline genetic polymorphisms of *STIM1* and TNM stage in estrogen receptor (ER) positive breast cancer patients.

dbSNV ID	Genotype	Late Stage ^a^ *N* (%)	Early Stage ^b^ *N* (%)	Genotype Model	
				OR (95% C.I.) ^c^	*p* Value
rs2304891	G/G	11 (33.3)	20 (14.4)	9.86 (2.47~39.39)	**9.18 × 10^−4^** **
	A/G	19 (57.6)	67 (48.2)	4.97 (1.39~17.75)	
	A/A	3 (9.1)	52 (37.4)	Reference	
rs3750996	G/G	0 (0)	10 (7.0)	0	**3.16 × 10^−3^** **
	A/G	5 (15.2)	51 (35.7)	0.28 (0.10~0.78)	
	A/A	28 (84.8)	82 (57.3)	Reference	
rs1561876	G/G	0 (0)	11 (8.1)	0	0.0909
	A/G	16 (53.3)	59 (43.7)	1.26 (0.56~2.80)	
	A/A	14 (46.7)	65 (48.1)	Reference	
rs3750994	G/G	1 (3.2)	5 (3.5)	0.87 (0.09~8.04)	0.9897
	T/G	11 (35.5)	52 (36.4)	0.97 (0.42~2.20)	
	T/T	19 (61.3)	86 (60.1)	Reference	

The *p* value was adjusted for age. ** *p* value < 0.005 are shown in bold. ^a^ Late-stage included stage III. ^b^ Early-stage included stages I and II. ^c^ Odds ratio (OR) and 95% confidence intervals (C.I.).

**Table 5:** Haplotype analysis of *STIM1* in early and late-stage breast cancer patients.

Haplotype ^a^	Frequency		OR (95% C.I.) ^d^	*p* Value ^e^
rs2304891/rs3750996/rs1561876/rs3750994	Late Stage ^b^	Early Stage ^c^		
G/A/A/T	0.548	0.367	3.84 (1.63~9.03)	0.0028 *
A/A/G/G	0.222	0.201	2.84 (1.11~7.28)	0.6631
A/A/G/T	0.065	0.066	2.74 (0.74~10.09)	0.9261
A/A/A/T	0.050	0.097	1.49 (0.41~5.37)	0.2104
A/G/A/T	0.088	0.230	Reference	Reference

* *p* value < 0.05 is shown in bold. ^a^ Haplotypes with a frequency of <1% were excluded. ^b^ Late-stage included stage III. ^c^ Early-stage included stages I and II. ^d^ Odds ratio (OR) and 95% confidence intervals (C.I.). ^e^ *p* values were calculated using an additive model.

## Supplementary Materials

The following are available online at https://www.mdpi.com/2075-4426/10/4/287/s1. Table S1: Association analysis between genetic polymorphisms of STIM1 and TNM stage in progesterone receptor (PR) positive breast cancer patients, Table S2: Association analysis between genetic polymorphisms of STIM1 and TNM stage in human epidermal growth factor receptor 2 (HER2) positive breast cancer patients, Table S3: Expression quantitative trait locus (eQTL) analysis of STIM1 SNVs, Figure S1: STIM1 gene expression among different genotypes of rs2304891 in (A) nerve-tibial tissue and (B) heart-left ventricle tissue.
