# Supplementary material for: Germline Genetic Association between Stromal Interaction Molecule 1 (STIM1) and Clinical Outcomes in Breast Cancer Patients
Source: J Pers Med. 2020 Dec 17;10(4):287. doi: 10.3390/jpm10040287 (PMC7767290; doi:10.3390/jpm10040287)
Supplement: Supplementary file 1 [file jpm-10-00287-s001.pdf]

Supplementary Table S1. Association analysis between germline genetic polymorphisms of *STIM1* and TNM stage in progesterone receptor (PR) positive breast cancer patients

dbSNV ID	Genotype	Late stage[a]	Early stage[b]	Genotype model	
		N (%)	*N* (%)	OR (95% C.I.)[c]	*p* value
rs2304891	G/G	8 (32.0)	17 (14.7)	10.51 (2.02~54.74)	**3.47×10^{-3}****
	A/G	15 (60.0)	55 (47.4)	6.02 (1.31~27.75)	
	A/A	2 (8.0)	44 (37.9)	Reference	
rs3750996	G/G	0 (0)	7 (5.9)	0	**8.53×10^{-3}***
	A/G	3 (12.0)	41 (34.7)	0.23 (0.07~0.82)	
	A/A	22 (88.0)	70 (59.3)	Reference	
rs1561876	G/G	0 (0)	10 (8.9)	0	0.1038
	A/G	14 (58.3)	51 (45.5)	1.40 (0.57~3.44)	
	A/A	10 (41.7)	51 (45.5)	Reference	
rs3750994	G/G	1 (4.3)	5 (4.2)	1.02 (0.11~9.79)	0.9826
	T/G	9 (39.1)	44 (37.3)	1.09 (0.43~2.78)	
	T/T	13 (56.5)	69 (58.5)	Reference	

The *p* value was adjusted for age. **p* value <0.05 and ***p* value <0.005 are shown in bold. [a]Late stage included stage III. [b]Early stage included stages I and II. [c]Odds ratio (OR) and 95% confidence intervals (C.I.).

Supplementary Table S2. Association analysis between germline genetic polymorphisms of *STIM1* and TNM stage in human epidermal growth factor receptor 2 (HER2) positive breast cancer patients

dbSNV ID	Genotype	Late stage[a]	Early stage[b]	Genotype model	
		N (%)	*N* (%)	OR (95% C.I.)[c]	*p* value
rs2304891	G/G	10 (34.5)	18 (16.4)	4.11 (1.21~13.92)	0.0618
	A/G	14 (48.3)	56 (50.9)	1.85 (0.61~5.59)	
	A/A	5 (17.2)	36 (32.7)	Reference	
rs3750996	G/G	0 (0)	6 (5.4)	0	**0.0315***
	A/G	5 (16.7)	36 (32.4)	0.38 (0.13~1.07)	
	A/A	25 (83.3)	69 (62.2)	Reference	
rs1561876	G/G	1 (3.8)	6 (5.6)	0.87 (0.09~8.29)	0.8979
	A/G	13 (50.0)	47 (43.9)	1.21 (0.50~2.92)	
	A/A	12 (46.2)	54 (50.5)	Reference	
rs3750994	G/G	2 (7.1)	2 (1.8)	5.17 (0.61~43.91)	0.3360
	T/G	9 (32.1)	37 (33.9)	0.97 (0.39~2.41)	
	T/T	17 (60.7)	70 (64.2)	Reference	

The *p* value was adjusted for age. **p* value <0.05 are shown in bold. [a]Late stage included stage III. [b]Early stage included stages I and II. [c]Odds ratio (OR) and 95% confidence intervals (C.I.).

Supplementary Table S3. Expression quantitative trait locus (eQTL) analysis of *STIM1* SNPs

Gene	dbSNP	alt allele	Tissue	p-value	NES[a]
STIM1	rs2304891	G	Nerve-Tibial	2.70×10^{-9}	0.17
STIM1	rs2304891	G	Heart-Left Ventricle	2.10×10^{-8}	0.22

[a]NES: Normalized effect size

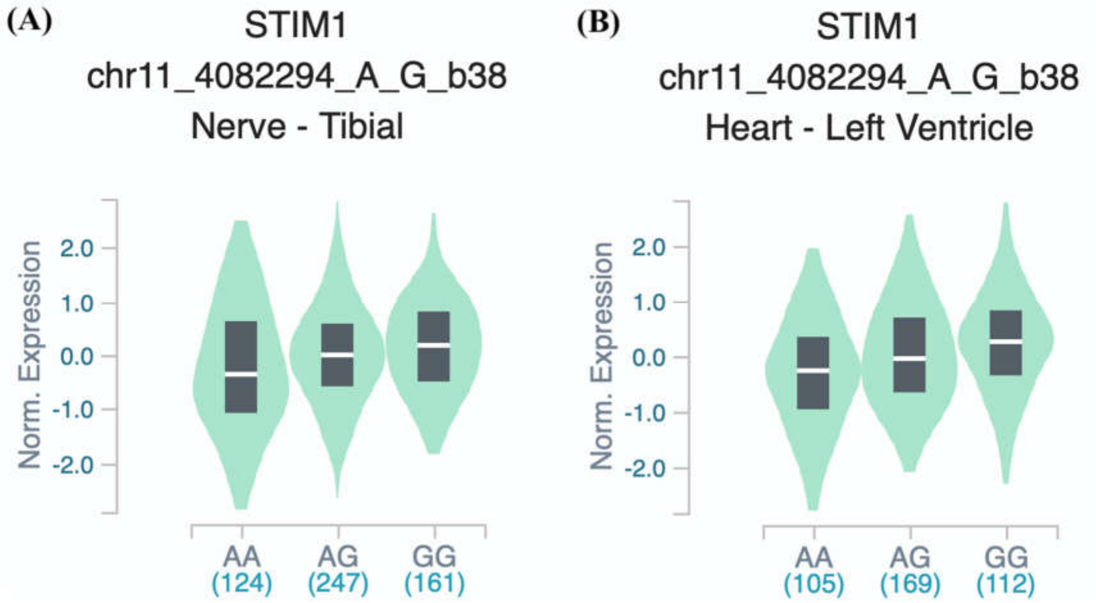

Supplementary Figure S1. *STIM1* gene expression among different genotypes of rs2304891 in (A) nerve-tibial tissue and (B) heart-left ventricle tissue.
